# Multi-institutional retrospective analysis of ultrahypofractionated radiotherapy for Japanese prostate cancer patients

**DOI:** 10.1038/s41598-021-92307-8

**Published:** 2021-06-23

**Authors:** Hiromichi Ishiyama, Hideyasu Tsumura, Hisato Nagano, Motoi Watanabe, Eiichi Mizuno, Masashi Taka, Hiroaki Kobayashi, Takahisa Eriguchi, Hajime Imada, Koji Inaba, Katsumasa Nakamura

**Affiliations:** 1 Department of Radiation Oncology, Kitasato University School of Medicine, 1-15-1 Kitasato, Minami-ku Sagamihara, Kanagawa Japan; 2 Department of Urology, Kitasato University School of Medicine, 1-15-1 Kitasato, Minami-ku Sagamihara, Kanagawa Japan; 3 Highly Accurate and Exact Radiation Therapy Center, Shonan Fujisawa Tokusyukai Hospital, 1-5-1 tsujido-kandai, Fujisawa, Kanagawa Japan; 4 Toyama CyberKnife Center, 1837-5 Hiyodorijima, Toyama, Japan; 5 Department of Radiation Therapy, Kouseiren Takaoka Hospital, 5-10 Eiraku-cho, Takaoka, Japan; 6 Department of Urology, Saiseikai Yokohamashi Tobu Hospital, 3-6-1 Shimosueyoshi, Tsurumi-ku, Yokohama, Kanagawa Japan; 7 Department of Radiation Oncology, Saiseikai Yokohamashi Tobu Hospital, 3-6-1 Shimosueyoshi, Tsurumi-ku, Yokohama, Kanagawa Japan; 8 Cancer Treatment Center, Tobata Kyoritsu Hospital, 2-5-1 Sawami, Tobata-ku, Kitakyusyu, Fukuoka Japan; 9 Department of Radiation Oncology, National Cancer Center Hospital, 5-1-1 Tsukiji, Chuo-ku, Tokyo, Japan; 10 Department of Radiation Oncology, Hamamatsu University School of Medicine, 1-20-1 Handayama, Higashi-ku, Hamamatsu, Shizuoka Japan

**Keywords:** Cancer, Urological cancer

## Abstract

To report outcomes and risk factors of ultrahypofractionated (UHF) radiotherapy for Japanese prostate cancer patients. This multi-institutional retrospective analysis comprised 259 patients with localized prostate cancer from 6 hospitals. A total dose of 35–36 Gy in 4–5 fractions was prescribed for sequential or alternate-day administration. Biochemical failure was defined according to the Phoenix ASTRO consensus. Toxicities were assessed using National Cancer Institute Common Toxicity Criteria version 4. Tumor control and toxicity rates were analyzed by competing risk frames. Median follow-up duration was 32 months (range 22–97 months). 2- and 3-year biochemical control rates were 97.7% and 96.4%, respectively. Initial prostate-specific antigen (p < 0.01) and neoadjuvant androgen deprivation therapy (p < 0.05) were identified as risk factors for biochemical recurrence. 2- and 3-year cumulative ≥ Grade 2 late genitourinary (GU) toxicities were 5.8% and 7.4%, respectively. Corresponding rates of gastrointestinal (GI) toxicities were 3.9% and 4.5%, respectively. Grade 3 rates were lower than 1% for both GU and GI toxicities. No grade 4 or higher toxicities were encountered. Biologically effective dose was identified as a risk factor for ≥ Grade 2 late GU and GI toxicities (p < 0.05). UHF radiotherapy offered effective, safe treatment for Japanese prostate cancer with short-term follow-up. Our result suggest higher prescribed doses are related to higher toxicity rates.

## Introduction

Ultrahypofractionated (UHF) radiotherapy is defined as > 5 Gy per fraction and has gradually been recognized as a standard treatment for localized prostate cancer. The biological features of prostate cancer with a low α/β ratio have encouraged widespread adoption of UHF radiotherapy around the world. In addition to these biological advantages, UHF radiotherapy offers benefits in terms of cost effectiveness and patient convenience. In addition, patients treated with UHF radiotherapy reported significantly “less regret” and “less toxicity” than expected compared to patients treated with other radiotherapy techniques^1^.

The current guidelines conditionally recommend UHF radiotherapy only for low- or intermediate-risk patients^2^. However, as the potential advantages of UHF radiotherapy over other treatment techniques are gradually confirmed, candidates for this treatment are expected to expand to not only low- and intermediate-risk patients, but also high-risk patients.

Meanwhile, the current state of UHF radiotherapy in Japan is unclear, although several Japanese institutions are likely to have already started UHF radiotherapy. In addition, treatment results for Japanese patients have not yet been reported except in a few papers^3–5^. We therefore conducted a survey of the current status of UHF radiotherapy in Japan and undertook a multi-institutional retrospective analysis of UHF radiotherapy for Japanese prostate cancer patients. We report herein the outcomes and risk factors of UHF radiotherapy in Japanese prostate cancer patients.

## Materials and methods

### Patients and treatments

We sent a questionnaire survey to around 160 Japanese institutions that were participants in the Japanese Radiation Oncology Study Group (JROSG) or that were equipped with CyberKnife systems between December 2019 and February 2020. The results showed that at least 10 institutions in Japan currently apply UHF radiotherapy for localized prostate cancer, and more than 1300 patients have already received treatment with UHF radiotherapy. Detailed results of the questionnaire survey are shown in the Supplementary Table S1.

Six of the 10 institutions agreed to participate in further retrospective analysis of patients treated with UHF radiotherapy. The Kitasato University Hospital institutional review board, Shonan Fujisawa Tokusyukai Hospital institutional review board, Toyama CyberKnife Center institutional review board, Saiseikai Yokohamashi Tobu Hospital institutional review board, Tobata Kyoritsu Hospital institutional review board, and National Cancer Center Hospital institutional review board approved the study protocol. Informed consent was obtained in the form of opt-out on the web-site. Those who rejected were excluded. This study was conducted in accordance with the Declaration of Helsinki.

Table 1 shows the background characteristics of patients. More than half of the patients (61%) were categorized as intermediate risk based on the National Comprehensive Cancer Network criteria. More than half of the patients (57%) had received neoadjuvant androgen deprivation therapy (ADT), and 32% had received adjuvant ADT. The mean (± standard deviation) duration of therapy was 6.5 (± 6.3) months for neoadjuvant ADT, and 12.5 (± 12.6) months for adjuvant ADT. The total dose was 35–36 Gy in 4–5 fractions, prescribed as sequential or alternate-day doses. The mean biologically effective dose (BED) based on α/β = 1.5 was 222.8 Gy. Most patients in our study population had no hydrogel spacer. Table 2 shows treatment protocols in the 6 participating institutions. Four of 6 institutions used CyberKnife. Five of 6 institutions used fiducial markers implanted in the prostate.

**Table 1:** Patient characteristics.

**Age, years**	72	(6.8)
**T**		
1a	2	
1b	1	
1c	96	
2a	83	
2b	22	
2c	30	
3a	14	
3b	11	
**Gleason score**		
5 + 5	7	
5 + 4	3	
4 + 5	19	
4 + 4	29	
4 + 3	51	
3 + 4	94	
3 + 3	56	
**iPSA**	12.1	(16.9)
**Risk criteria**		
Low	33	
Intermediate	158	
High	68	
**Neo ADT**		
Yes	147	
No	112	
**Adj ADT**		
Yes	83	
No	176	
**Total dose**	35.6	(0.9)
**Number of fractions**	4.6	(0.5)
**BED**	222.8	(22.5)
**Hydrogel spacer**		
Yes	31	
No	228	

*ADT* androgen deprivation therapy; *iPSA* initial prostate cancer-specific antigen; *GS* Gleason score; *BED* biologically effective dose. Values are number or mean (standard deviation).

**Table 2:** Treatment protocols.

Institution	Dose per fraction	Number of fractions	Prescription	Schedule	Target	Treatment system	Fiducial marker	Urethral catheter
1	7.25 Gy	5	D95	Sequential	CTV = prostate + (SV 1 cm + 3 mm (posterior 1 mm)); PTV = CTV + 2 mm	Cyberknife	Yes	No
2	7.25 Gy	5	D95	Sequential	Low risk: CTV = GTV + 3 mm; PTV = CTV + 2 mm Intermediate risk: CTV = GTV + 3 mm; PTV = CTV + 2 mm	Cyberknife M6	Yes	No
3	7.25 Gy	5	D83	Alternate-day	Low risk: CTV = prostate; PTV = CTV + 5 mm (posterior 3 mm) Intermediate risk: CTV = prostate + SV 1 cm; PTV = CTV + 5 mm (posterior 3 mm) High risk: CTV = prostate + SV 1–2 cm; PTV = CTV + 5 mm (posterior 3 mm)	Cyberknife	Yes	No
4	8–9 Gy	4	D95	Sequential (2-day break)	CTV = prostate + SV 1 cm; PTV = CTV + 5 mm (posterior 3 mm)	Tomotherapy	Yes	Yes
5	7 Gy	5	D98-99	Sequential Alternate-day Twice-weekly	Low risk: CTV = prostate; PTV = CTV + 3 mm Intermediate risk: CTV = prostate + proximal SV; PTV = CTV + 3 mm high Risk: CTV = prostate + SV; PTV = CTV + 3 mm	Liniac	No	No
6	7.25 Gy	5	D95	Alternate-day	low Risk: CTV = prostate; PTV = CTV + 5 mm (posterior 4 mm) Intermediate risk: CTV = prostate + half of SV; PTV = CTV + 5 mm (posterior 4 mm)	Cyberknife Liniac	Yes	No

*CTV* clinical target volume, *SV* seminal vesicle, *PTV* planning target volume.

### Statistical analysis

Statistical analyses were performed using R version 3.5.1 software (R Project for Statistical Computing, Vienna, Austria). Overall survival was calculated using the Kaplan–Meier method. Biochemical failure was defined according to the Phoenix ASTRO consensus (Nadir + 2)^6^. Genitourinary (GU) and gastrointestinal (GI) toxicities were assessed using the National Cancer Institute Common Toxicity Criteria version 4.

A competing risk analysis (Gray’s test and Fine and Gray’s regression) was used for biochemical control, local recurrence, pelvic lymph-node recurrence, distant metastasis, castration-resistant prostate cancer (CRPC) and cumulative GU and GI toxicity rates. Crude rates of GU and GI toxicities are also reported for comparison with other reports. Age, T stage, Gleason score, initial prostate-specific antigen (PSA), BED, neoadjuvant ADT, and adjuvant ADT were included as variates in univariate analyses. Multivariate models for each endpoint were constructed by including all factors with values of p < 0.20 from univariate analyses. Values of p < 0.05 were considered statistically significant.

### Ethics approval and consent to participate

This study was approved by the local institutional review boards.

### Consent for publication

Informed consent was obtained in the form of opt-out on the web-site. Those who rejected were excluded.

## Results

**Figure 1:**
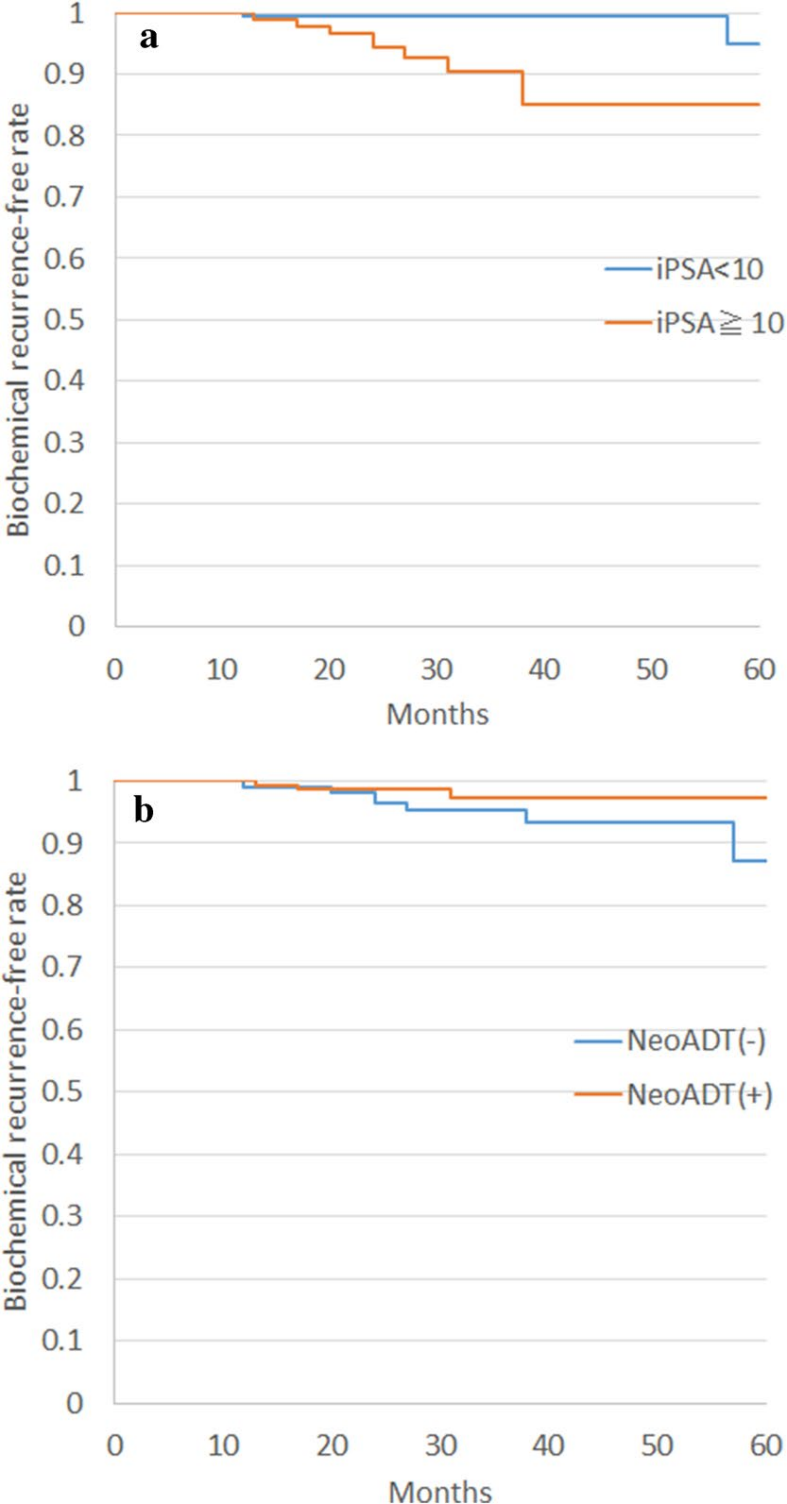
Differences in biochemical recurrence-free rates depend on initial PSA value (**a**) and neoadjuvant androgen deprivation therapy (**b**).

The median duration of follow-up was 32 months (range 22–97 months). Two- and 3-year overall survival rates were 99.6% (95% CI 0.99–100%) and 99.1% (95% CI 97.9–100%), respectively. No prostate cancer deaths were reported. Two- and 3-year biochemical control rates were 97.7% (95% CI 95.2–99.0%) and 96.4% (95% CI 93.3–98.4), respectively. Corresponding rates of each risk category were as follows: low risk, 100% and 100% (95% CI na); intermediate risk, 97.5% (95% CI 94.0–99.2%) and 96.6% (95% CI 92.7–98.7%); and high risk, 97.1% (95% CI 90.8–99.5%) and 93.7% (95% CI 83.2–98.6%), respectively. Initial PSA and neoadjuvant ADT were detected as risk factors for biochemical recurrence by multivariate analysis (Table 3, Fig. 1).

**Table 3:** Uni- and multivariate analyses of biochemical relapse-free rate.

Risk factor	Univariate			Multivariate		
	HR	95% CI	p value	HR	95% CI	p value
**Age**	0.96	(0.88–1.05)	0.78			–
**iPSA**						
< 10	Ref		0.00	Ref		0.00
≧10	10.1	(2.30–44.40)		10.70	(2.69–42.89)	
**GS**	1.45	(0.75–2.81)	0.51			–
**T stage**						
< 3a	Ref		0.72			–
≧3a	1.45	(0.18–11.70)				
**Neoadjuvant ADT**						
No	Ref		0.14	Ref		0.03
Yes	0.37	(0.01–1.43)		0.21	(0.05–0.85)	
**Adjuvant ADT**						
No	Ref		0.57			–
Yes	0.63	(0.13–3.04)				
**BED**	1.02	(1.00–1.05)	0.10	1.02	(1.00–1.05)	0.15

*iPSA* initial prostate cancer-specific antigen, *GS* Gleason score; *BED* biologically effective dose; *ADT* androgen deprivation therapy.

Two-year local recurrence, pelvic lymph-node recurrence, and distant metastasis rates were 0.4%, 0.4%, and 1.2%, respectively. Corresponding rates at 3 years were 1.3%, 0.4%, and 1.8%, respectively. Two- and 3-year CRPC rates were 0.4% and 1%, respectively.

Table 4 shows crude rates of acute and late toxicities. Grade 3 toxicity rate was lower than 1%. No grade 4 or higher toxicity was seen during follow-up. The most common acute ≥ Grade 2 GU toxicities were frequency (17/37, 46%), retention (13/37, 35%), and pain (3/37, 4%). Corresponding GI toxicities were diarrhea (7/13, 54%), proctitis (3/13, 23%), and bleeding (3/13, 23%). The most common late > Grade 2 GU toxicities were frequency (8/18, 44%), retention (2/18, 11%), and pain (2/18, 11%). The corresponding GI toxicity was bleeding (10/12, 83%).

**Table 4:** Toxicity rates.

	Acute GU		Acute GI		Late GU		Late GI	
Grade 1	95	(36.7%)	55	(21.2%)	71	(27.4%)	36	(13.9%)
Grade 2	36	(13.9%)	13	(5.0%)	16	(6.2%)	10	(3.9%)
Grade 3	1	(0.4%)	0	(0.0%)	2	(0.8%)	2	(0.8%)

*GU* genitourinary. *GI* gastrointestinal.

**Figure 2:**
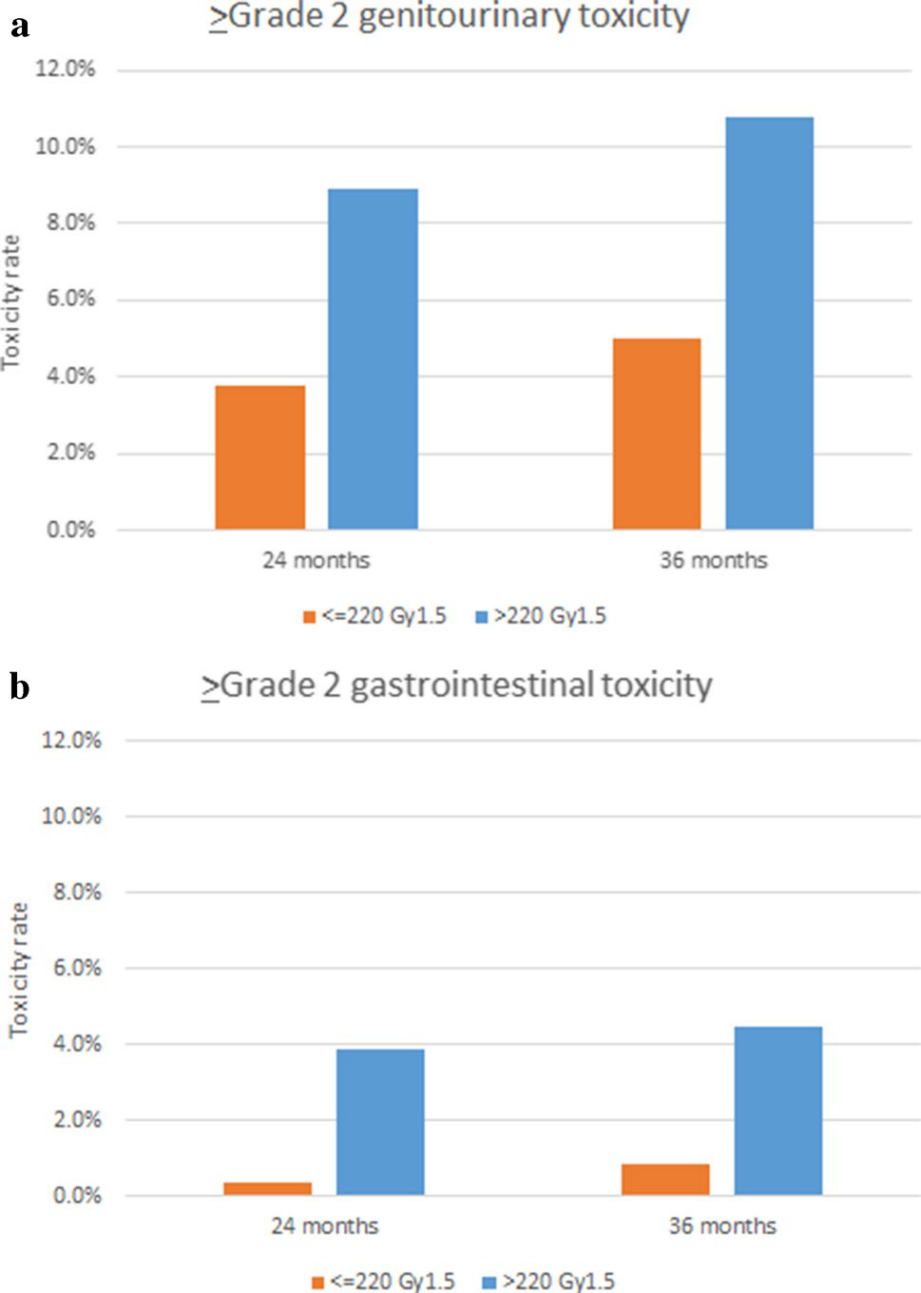
Crude toxicity rates for Grade ≥ 2 genitourinary (**a**) and gastrointestinal, (**b**) toxicities depend on biologically equivalent doses (> 220 or ≤ 220 Gy_1.5_).

2- and 3-year cumulative ≥ Grade 2 late GU toxicity rates were 5.8% and 7.4%, respectively. Corresponding rates of GI toxicities were 3.9% and 4.5%, respectively. BED was detected as a risk factor for ≥ Grade 2 late GU and GI toxicities (Table 5, Fig. 2). No other variables was detected with values of p < 0.20 in univariate analyses.

**Table 5:** Univariate analyses of > Grade 2 late toxicity.

Risk factor	Late GU toxicity			Late GI toxicity		
	HR	95% CI	p value	HR	95% CI	p value
**Age**	0.96	(0.89–1.03)	0.24	1.02	(0.93–1.12)	0.66
**iPSA**						
< 10	Ref		0.31	Ref		0.55
≧10	1.65	(0.63–4.30)		1.41	(0.45–4.40)	
**GS**	1.20	(0.77–1.89)	0.42	1.07	(0.57–2.00)	0.84
**T stage**						
< 3a	Ref		0.65	Ref		0.96
≧3a	0.63	(0.08–4.72)		0.95	(0.13–7.25)	
**Neoadjuvant ADT**						
No	Ref		0.57	Ref		0.71
Yes	1.31	(0.51–3.39)		0.80	(0.25–2.60)	
**Adjuvant ADT**						
No	Ref		0.82	Ref		
Yes	0.89	(0.32–2.48)		0.44	(0.10–2.01)	0.29
**BED**	1.02	(1.00–1.04)	0.03	1.03	(1.00–1.07)	0.03

*GU* genitourinary, *GI* gastrointestinal, *iPSA* initial prostate cancer-specific antigen, *GS* Gleason score, *BED* biologically effective dose, *ADT* androgen deprivation therapy.

## Discussion

Our retrospective analysis showed that the UHF radiotherapy is safe and effective for Japanese patients, at least in a short-term follow-up. Considering recently reported long-term^7^ and large cohort^8^ outcomes from Western countries, this treatment is also valuable as a treatment option for Japanese prostate cancer patients. Although a cautious approach is warranted until long-term results become available, there are no obstacles to applying UHF radiotherapy for Japanese patients.

Our analysis revealed that prescription doses were significantly related to toxicity rates. The American Society for Radiation Oncology (ASTRO), American Society of Clinical Oncology (ASCO), and American Urological Association (AUA) guidelines recommend prescription doses between 35 Gy and 36.25 Gy in 5 fractions, and doses above 36.25 Gy are not suggested outside the setting of clinical trials due to the risk of late toxicities^2^. Table 6 shows the results of previous reports of relatively high incidences of ≥ Grade 2 toxicities^7,9–16^. Although it was not always high-dose prescriptions that led to high toxicity rates in previous studies, prescribed doses seem related to more severe toxicity, as our study suggested. Because our study included patients who participated in dose-escalation trials testing relatively high doses, these patients suffered relatively severe late toxicities compared to patients treated with current standard doses following the guidelines^4^.

**Table 6:** Selected series reported > 5% Grade 2 toxicities.

Author	Year	Schedule	BED α/β = 1.5	Median follow-up	Late GU	Late GI
Aluwini	2013	38 Gy/4 fr	278.7 Gy	28.35 months	G2: 15% G3: 5%	G2: 3%
Chen	2013	35–36.5 Gy/5 fr	198–211.5 Gy	27.6 months	G2: 31% (2-year)	G2: 1% (2-year)
Meier	2018	40 Gy/5 fr	253.3 Gy	61 months	G2: 12% G3: 1.3%	G2: 2%
Katz	2017	35–36.5 Gy/5 fr	198–211.5 Gy	108 months	G2: 9% G3: 3%	G2: 4%
Zimmermann	2016	45 Gy/9 fr	315 Gy	83 months	G2: 27.5% G3: 2.5% G4: 1.3%	G2: 17.5% G3: 12.5%
Kim	2014	45–50 Gy/5 fr	315–383.3 Gy	24.5 months	NA	G4: 2.2% G3: 3.3% G2: 23.1%
Bernetich	2014	35–37.5 Gy/5 fr	198–225 Gy	38 months	G2: 14% G3: 2%	G2: 3%
Zhang	2017	38 Gy/4 fr	278.7 Gy	35.5 months	G2: 19.2% G3: 2.6%	NA
Helou	2017	35–40 Gy/5 fr	198–253.3 Gy	38 months	G3: 1.9% G2: 32.6%	G2: 12.0% G3: 0.8% G4: 1.1%

*BED* biologically effective dose, *GU* genitourinary, *GI* gastrointestinal.

Koontz et al. published a review analyzing data from pioneering institutions and reported that over 100 Gy-equivalents in 2-Gy fractions might cause higher rates of > Grade 2 toxicities^17^. Because the schedule of 36 Gy/4 fractions included in our study was equal to 108 Gy-equivalents in 2-Gy fractions and a BED of 226 Gy, our results appear consistent with their suggestion.

From the perspective of toxicity, a lower prescribed dose might be suitable, especially for low- and favorable intermediate-risk patients. However, a lower dose might cause lower tumor control, and the importance of dose escalation is well known in conventional fractionation^18,19^. In the field of UHF radiotherapy, Zelefsky et al. suggested the importance of dose escalation. Although long-term control rates were not determined, they reported positive biopsy rates of 47.6%, 19.2%, 16.7% and 7.7% after 32.5 Gy, 35 Gy, 37.5 Gy and 40 Gy in 5 fractions, respectively^20^. These results suggest that unfavorable tumor control rates might be seen in lower-dose groups.

Helou et al. reported that the 3-year PSA level correlated with the prescribed dose in a comparison between 40 Gy (0.27 ng/ml) and 35 Gy (0.64 ng/ml) in 5 fractions. The higher dose of 40 Gy was an independent predictor of a lower 3-year PSA level in their multivariate analysis^16^. The 3-year PSA value was previously found to offer an early predictor of biochemical failure after high-dose-rate brachytherapy^21^. The concept of a well-balanced, optimal dose during UHF radiotherapy thus remains contentious and should be explored in future trials.

Meanwhile, periprostatic hydrogel spacers have been approved for use with transperineal injection in Japan since 2017. Current practices in UHF radiotherapy are thus expected to usually be combined with spacer injection, and may decrease severe toxicities even using the same dose levels^22^.

Regarding risk factors for biochemical recurrence, our results were not surprising from the point of view of experience with conventional external beam radiotherapy (EBRT). The nomogram established by Kattan et al. more than 20 years ago revealed that the initial PSA level was the most powerful indicator of biochemical recurrence^23^. ADT is well known as another powerful indicator of biochemical recurrence^24^. Our results thus suggested that UHF radiotherapy shows similar trends to conventional EBRT, although our short follow-up makes the value of these risk factors difficult to confirm.

Because our study was a retrospective analysis, several limitations should be considered. First, as collected items were limited, other factors might correlate with tumor control and toxicity rates. Second, our analysis of toxicities might have been biased due to the lack of information on dose-volume histograms. Third, because various treatment schedules were used at each hospital, results reported in this paper would have varied depending on treatment schedules. Fourth, because no follow-up schedule was defined, the timings of follow-up visits and examinations among hospitals were highly heterogeneous. Tumor control and toxicity rates might thus be relatively ambiguous.

## Conclusion

This multi-institutional analysis showed that UHF radiotherapy is effective for Japanese prostate cancer with limited severe toxicity. However, the optimal dose during UHF radiotherapy should be continuously explored, as our results also suggested that higher prescribed doses were related to higher toxicity rates.

## Supplementary Information

Supplementary Information.

## Data Availability

The datasets used and/or analysed during the current study are available from the corresponding author on reasonable request.

## Abbreviations

UHF: Ultrahypofractionated
GU: Genitourinary
GI: Gastrointestinal
ASTRO: American Society for Radiation Oncology
JROSG: Japanese Radiation Oncology Study Group
ADT: Androgen deprivation therapy
CRPC: Castration-resistant prostate cancer
PSA: Prostate-specific antigen
BED: Biologically effective dose
ASCO: American Society of Clinical Oncology
AUA: American Urological Association
EBRT: External beam radiotherapy
