# Supplementary material for: Multi-institutional retrospective analysis of ultrahypofractionated radiotherapy for Japanese prostate cancer patients
Source: Sci Rep. 2021 Jun 23;11:13194. doi: 10.1038/s41598-021-92307-8 (PMC8222240; doi:10.1038/s41598-021-92307-8)
Supplement: Supplementary file 1 [file 41598_2021_92307_MOESM1_ESM.docx]

| | |
|---|---|
| Q13 | Do you insert a baloon catheter for treatment? |
